# Preoperative magnetic resonance imaging for detecting uni- and bilateral extraprostatic disease in patients with prostate cancer

**DOI:** 10.1007/s00345-014-1362-x

**Published:** 2014-07-25

**Authors:** Erik Rud, Dagmar Klotz, Kristin Rennesund, Eduard Baco, Truls Erik Bjerklund Johansen, Lien My Diep, Aud Svindland, Lars Magne Eri, Heidi B. Eggesbø

**Affiliations:** Oslo University Hospital, Oslo, Norway

**Keywords:** Prostate cancer, MRI, Staging, Extraprostatic disease, Accuracy

## Abstract

**Objective:**

The objective of the study was to evaluate the diagnostic accuracy of preoperative magnetic resonance imaging (MRI) for detecting uni- and bilateral extraprostatic disease (T3) in patients with prostate cancer (PCa).

**Materials and methods:**

This prospective study included 199 patients with biopsy-proven PCa who underwent MRI prior to radical prostatectomy from December 2009 to July 2012. Extraprostatic extension and seminal vesicle invasion represented T3 disease, and was classified as uni- (right or left) or bilateral. MRI detection of T3 disease was assessed by descriptive statistics and odds ratio (OR). Whole-mount histopathology was used as the reference standard.

**Results:**

The overall prevalence of pT3 was 105/199 (53 %), unilateral in 81/105 (77 %) and bilateral in 24/105 (23 %). The sensitivity of MRI for predicting pT3 was 76/105 (72 %), specificity 61/94 (65 %), accuracy 137/199 (69 %), and OR 4.8 (95 % CI 2.7–8.8). A complete match with respect to the laterality of pT3 was found in 52/105 (50 %), and the side-specific accuracy was 113/199 (57 %). When unilateral pT3 was found, MRI falsely suggested contralateral T3 in 4/81 (5 %) and bilateral in 8/81 (10 %). When bilateral pT3 was found, MRI falsely suggested unilateral T3 in 12/24 (50 %).

**Conclusion:**

Magnetic resonance imaging (MRI) detected 72 % of all patients with T3 disease, and the accuracy dropped from 69 to 57 % when considering the laterality of T3. Thus far, the MRI technique is not yet adequate to meet the increasing demands of accurate diagnosis of locally advanced disease, and the contemporary MRI staging should be careful.

## Background

Accurate staging of prostate cancer (PCa) is essential for treatment planning. Digital rectal examination (DRE) still serves as the criterion standard for clinical tumor staging (cT), although known to be highly inaccurate [1, 2]. Extraprostatic extension (EPE) and/or seminal vesicle invasion (SVI) are criteria for extraprostatic disease, classified as T3. These patients have traditionally been treated with external beam radiation therapy (EBRT) and/or hormonal therapy. Today, surgery is increasingly being performed, and more sensitive methods for detecting T3 disease are desirable.

During the past decade, magnetic resonance imaging (MRI) has shown promising results for detecting PCa [3]. However, detecting T3 disease remains challenging, and the sensitivity and specificity range from 23 to 90 % and 30 to 95 %, largely dependent upon patient selection and the method used [4–10]. Furthermore, no MRI studies have stratified the detection of T3 disease according to uni- or bilateral condition, which is highly relevant when planning treatment. Nerve-sparing surgery or focal therapy would most probably be avoided on the same side as MRI suggests T3.

The aim of this study was to evaluate the diagnostic accuracy of preoperative MRI for detecting uni- and bilateral extraprostatic disease.

## Materials

This prospective study included 199 patients with biopsy-proven PCa between December 2009 and July 2012. The cohort constituted the MRI arm of a clinical trial where all patients were consecutively randomized to MRI or not, prior to robot-assisted laparoscopic prostatectomy (RALP). The study was approved by the Regional Ethical Committee (S-09143c2009/2183) and registered at ClinicalTrial.gov (NCT01347320). All patients had signed a letter of informed consent.

The following clinical features characterized the patients; cT1: 110 (55 %), cT2: 82 (41 %), and cT3: 7 (4 %). Gleason score (GS) in biopsy: 6: 64 (32 %), 7: 102 (51 %), 8: 25 (13 %), and 9: 8 (4 %). GS in specimen: 6: 53 (26 %), 7: 120 (60 %), 8: 19 (10 %), and 9: 7 (4 %). D’Amico risk classification low: 53 (27 %), intermediate: 101 (51 %), and high: 45 (23 %).

The mean age ± SD was 64 ± 6 years (range 42–74), and their prostate volume was 37 ± 16 mL (range 9–94). The median PSA ± interquartile range was 7.8 ± 5.6 ng/mL (range 1.2–54.0). The clinical and pathological data are displayed in Table 1.

**Table 1:** The overall detection of extraprostatic disease in 199 patients

		*n*	Sens	95 % CI	Spec	95 % CI	NPV	95 % CI	PPV	95 % CI	OR	95 % CI
MRI	All patients	199	72	63–81	65	54–75	68	57–77	70	60–78	4.8	2.7–8.8
	D’Amico risk group											
	Low	53	68	45–86	67	49–83	75	55–89	60	39–79	4.5	1.4–14.5
	Med	101	67	53–79	61	45–75	61	45–75	67	53–79	3.3	1.4–7.2
	High	45	86	67–96	71	44–90	75	48–93	83	64–95	14.4	3.3–63.6
DRE	All patients	199	5	2–11	98	92–99	48	41–55	71	30–95	2.3	0.4–12.1

*NPV* negative predictive value, *PPV* positive predictive value, *OR* odds ratio, *CI* confidence interval, *DRE* digital rectal examination

The MRI tumor detection rate and cancer volume estimation of this cohort have been reported previously [11].

## Methods

Magnetic resonance imaging (MRI) examinations were performed using a 1.5T Avanto system (Siemens, Erlangen, Germany) fitted with a six-channel body matrix coil (Siemens). MRI sequences included 3D T2-weighted images using 0.9-mm isotropic voxels and diffusion-weighted (DW) images with b50, b1000, and b2000. The apparent diffusion coefficient (ADC) map was calculated from b50 and b1000. Whole-mount histopathology sections were prepared perpendicular to the urethra and compared to the axial MRIs. MRI technique, surgical procedure, and specimen handling have previously been described in detail [11]. The time interval (median ± interquartile range) between biopsy and MRI was 11 ± 7 weeks (range 0–71), and between MRI and surgery was 1 ± 4 weeks (range 0–25).

All patients were classified as having either localized (T2), or locally advanced disease (T3) on MRI, DRE, and histopathology. EPE and/or SVI constituted T3 disease, and was classified as uni- (right or left) or bilateral. Whole-mount histopathology was used as the reference standard.

At DRE, clinically localized cancer included cT1 and cT2, while cT3 represented extraprostatic disease.

At MRI, EPE was defined as bulging of the capsule, irregular or ill-defined capsular surface, thickened neurovascular bundle, or visible invasion of the bladder neck [12–14]. Indirect signs of EPE, such as length of tumor capsular contact and tumor volume, were used for assessment, although no cutoff values were defined prior to the study. SVI was defined as an expansion with low T2 signal and reduced diffusion capacity, and obliteration of the angle between the prostate base and the vesicles [14].

At histopathology, EPE was defined as any visible tumor cells outside the prostate boundaries according to the guidelines from the International Society of Uropathologists (ISUP) Consensus Conference 2010 [15]. Bladder neck invasion was defined as T3. In the base and extreme apex, where capsule is sparse or barely present, EPE was diagnosed when the cancer extended into the periprostatic fatty tissue. SVI was defined as tumor cells present in the muscular layer according to ISUP consensus [16].

The length of EPE was defined as the sum of the circumferential diameter of EPE, measured at maximum two sites. The diameter was measured in the axial plane on the whole-mount histology sections and stratified according to detected and non-detected at MRI.

One radiologist (E.R.) with six years accumulated experience performed all MRI interpretations, and the histopathological staging was performed by an experienced (>20 years) uropathologist. Another resident pathologist (D.K.) reevaluated all specimens and performed the correlation analyses together with the radiologist. All pathologists were blinded to the MRI findings. The radiologist was not systematically blinded to clinical parameters such as cT, PSA, or biopsy results, although they were only occasionally known.

## Statistical analyses

Descriptive statistics and odds ratios (ORs) for DRE and MRI were calculated for predicting T3 disease using whole-mount histopathology as a reference standard. ORs were considered significant when the confidence interval did not include the value 1. The side-specific accuracy was defined as the patients with the correct side-specific diagnosis of T3 (right, left, bilateral) plus the number of true positive T2 divided by all patients. The results were stratified according to D’Amico risk groups [17]. The difference in length of detected and non-detected EPE was assessed by Mann–Whitney *U* test. Furthermore, patients with T3 were divided into two groups defined by ± the median length of EPE. Any difference in MRI detection was assessed by two-sided Pearson’s chi-square test (*p* < 0.05 was considered significant). MedCalc version 13.0.2 and IBM SPSS version 21 software were used for descriptive analysis and statistical tests.

## Results

The overall prevalence of pT3 disease was 105/199 (53 %), unilateral in 81/105 (77 %) and bilateral in 24/105 (23 %). The overall MRI sensitivity for detecting T3 was 76/105 (72 %), and the accuracy was 137/199 (69 %). The sensitivity of DRE for detecting T3 was 5/105 (5 %), specificity 92/94 (98 %), and accuracy 97/199 (49 %). It was five times more likely to be pT3 when T3 was suggested at MRI (OR = 4.8), whereas DRE could not predict pT3. The MRI and DRE detection of T3 disease is summarized in Table 1.

**Fig. 1:**
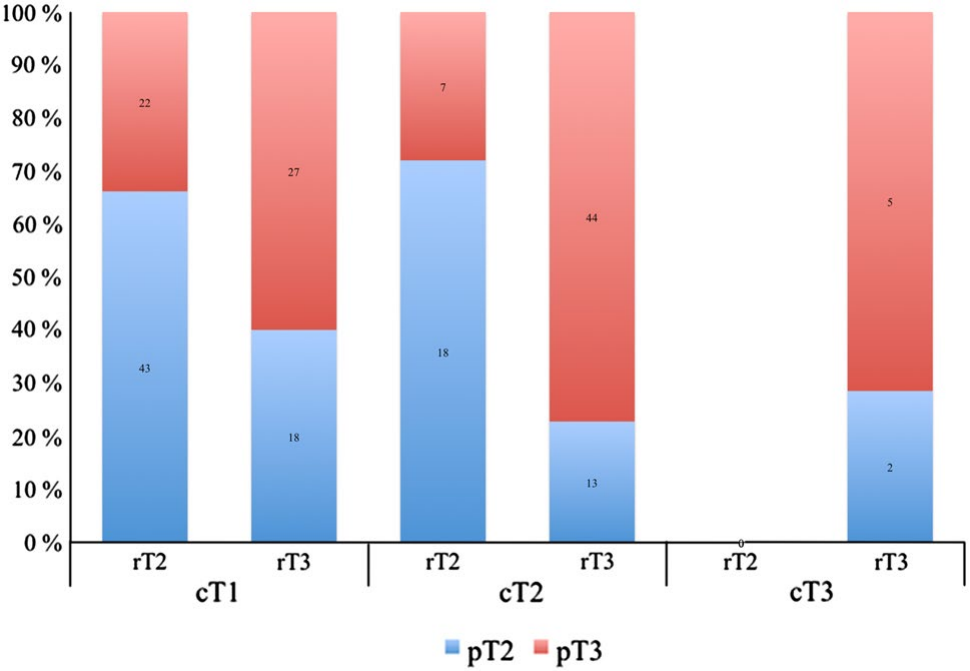
Illustrates the correlation between clinical, radiological, and pathological T classification. In patients with cT1, the sensitivity for predicting T3 disease was 55 %, specificity 70 %, and accuracy 64 %. Moreover, in patients with cT2, the sensitivity was 86 %, specificity 58 %, and accuracy 76 %. cT, rT, and pT: clinical, radiological, and pathological T classification

No patients were down-staged at MRI. In patients with cT1 and cT2, the accuracy of MRI for predicting T3 was 70/110 (64 %) and 62/44 (76 %), respectively. Figure 1 illustrates the correlation between clinical, radiological, and pathological T classification.

**Fig. 2:**
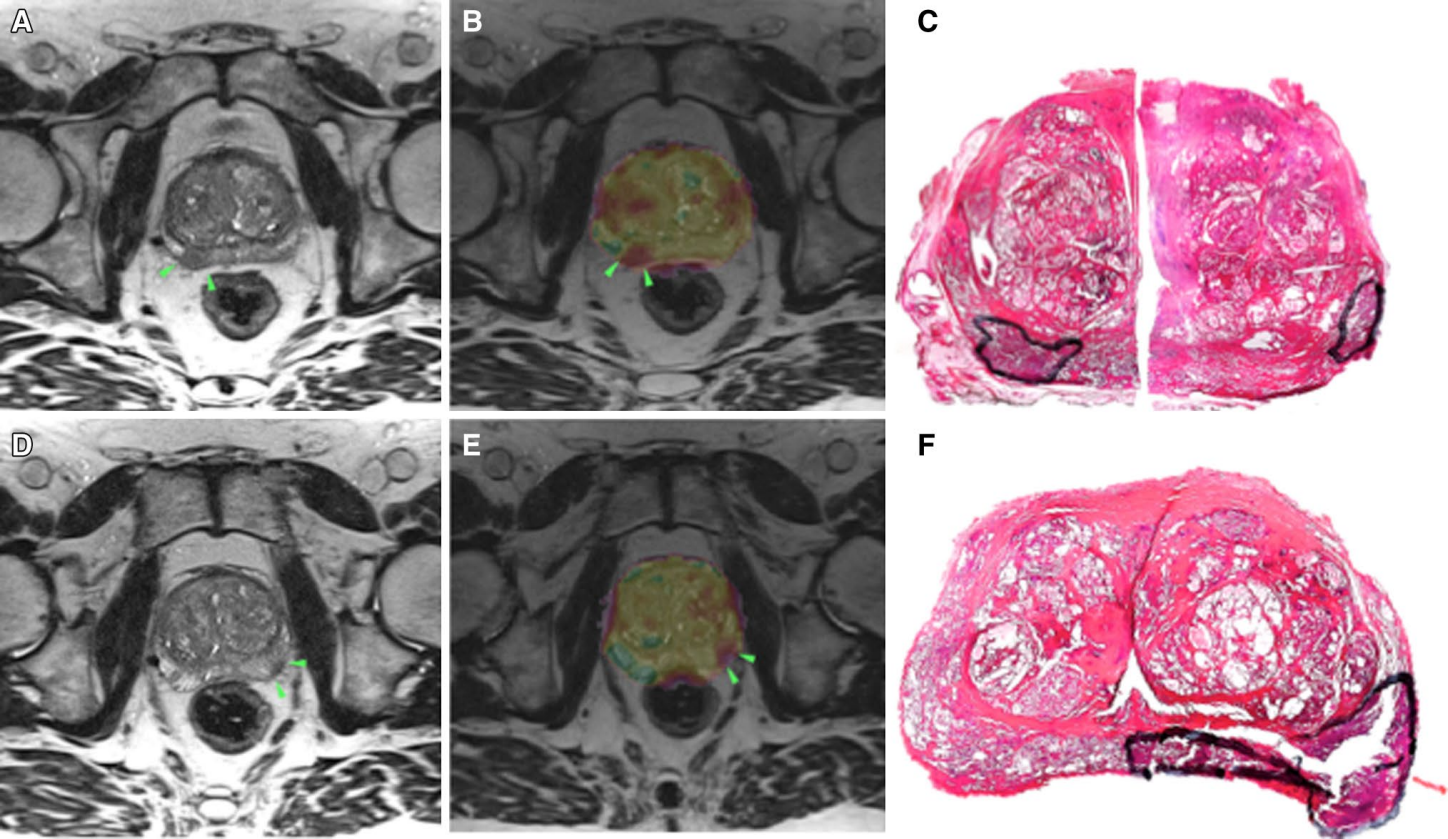
A 63-year-old patient with PSA 14.0 ng/mL, cT1c, and biopsy GS 7a. Axial T2-weighted images (**a**, **c**, **d**, **f**) and axial ADC color overlays (**b**, **e**) demonstrate tumors on both sides (*green arrowheads*). MRI suggested right-sided EPE. Whole-mount histopathology (**c**, **f**) verified the existence of both tumors, although pEPE was present on the *left side*. The surgical margins were negative

**Fig. 3:**
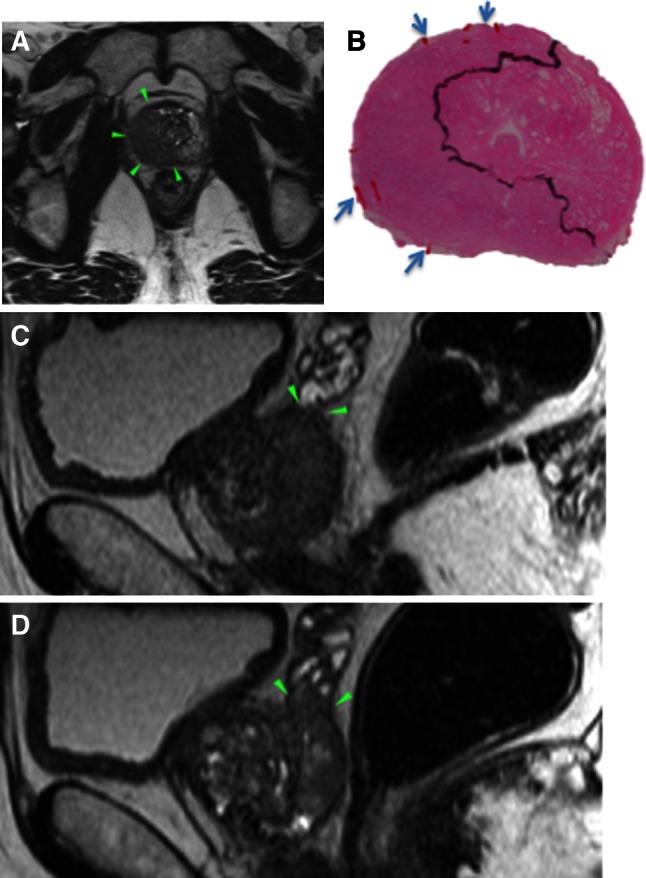
A 62-year-old patient with PSA 5.8 ng/mL, cT2, and biopsy GS 8. Axial T2-weighted image (**a**) demonstrates a large tumor affecting both lobes, although dominating the *right side*. *Right-sided* EPE and bilateral SVI (*green arrowheads* in **c** and **d**) were suggested at MRI, e.g., bilateral T3. Histopathology confirmed *right-sided* pEPE (**b**) (*blue arrows*), although no pSVI was present. The surgical margins were positive on the *right side*

A complete match with respect to the laterality of T3 was found in 52/105 (50 %), and the side-specific accuracy was 113/199 (57 %). When unilateral pT3 was found, MRI falsely suggested contralateral T3 in 4/81 (5 %) (Fig. 2) and bilateral T3 in 8/81 (10 %) (Fig. 3). When bilateral pT3 was found, MRI falsely suggested unilateral T3 in 12/24 (50 %). The side-specific agreement of T3 disease between MRI and histopathology is presented in Table 2.

**Table 2:** The side-specific agreement of T3 between MRI and histopathology

		T3 at MRI							
		Total		Negative		Unilateral		Bilateral	
		*n*	%	*n*	%	*n*	%	*n*	%
pT3	Negative	94	47	61	65	27	29	6	6
	Unilateral	81	41	26	32	47*	58	8	10
	Bilateral	24	12	3	13	12	54	9	38
	Total	199	100	90	45	86	43	23	12

* Forty-three patients had ipsilateral T3 at MRI, and four had contralateral T3

The positive predictive value (PPV) was highest for D’Amico high-risk patients (83 %), while the highest negative predictive value (NPV) was 75 % for both low- and high-risk patients (Table 1).

The length (median ± interquartile range) of EPE was 8.0 ± 9 mm. In patients correctly classified as T3, the length was 9.5 ± 8 mm, compared to 6.0 ± 9 mm in those erroneously classified as T2 (*p* = 0.03). MRI detected 40/49 (82 %) of those with EPE >8 mm and 34/51 (67 %) of those with EPE <8 mm (*p* = 0.09). The length of EPE was missing in five patients.

## Discussion

This study demonstrated 72 % overall sensitivity for detecting T3 disease, compered to 43 and 58 % recently described in two other publications [5, 6]. However, when accounting the laterality of T3 in our study, the accuracy dropped from 69 to 57 %. To the contrary, Cornud et al. [12] (prospective, *n* = 178) did not report any side-specific inconsistency, though no patients had bilateral pT3, and the overall prevalence was low compared to ours (21 vs. 51 %). To our knowledge, all other studies report EPE in each lobe and SVI on each side separately, making comparison difficult [4–9]. Such simplification masks bilateral T3 disease, which is highly relevant when planning both focal treatment and nerve-sparing surgery.

Mediocre results for detecting T3 disease in unselected patient cohorts may be important reasons why preoperative MRI has not yet proved its role. However, since DRE cannot predict T3 to a significant level, MRI is still the best method available [1, 2, 5]. Furthermore, MRI performs well in selected patient groups, such as high-risk patients and patients with cT2 (Table 1; Fig. 1). In both these groups, MRI demonstrated 86 % sensitivity for detecting T3, which might be clinically important. A few studies report a positive impact of preoperative MRI with regard to preserving or resecting the neurovascular bundle (NVB) in selected patients [18–20]. By contrast, Brown et al. [21] (retrospective, *n* = 62) reported a possible negative impact of MRI upon the surgical margin status. For these, and other reasons, MRI is still not recommended prior to surgery.

The predictive values may be important when assessing the likelihood of having T3 disease in the individual patient, and the highest PPV (83 %) was found in high-risk patients (Table 1). However, the clinical impact of a high PPV in these patients is possibly minor, since they would rarely undergo nerve-sparing surgery regardless of MRI. On the contrary, there is a moderately high NPV in low-risk patients (75 %), and in this situation, MRI may favor nerve-sparing surgery or active surveillance. Somford et al demonstrated both higher NPV and PPV for low- and high-risk patients, respectively [5]. The Partin’s nomogram is often used for predicting the likelihood of T3, although a recent study by Gupta et al. [22] (retrospective, *n* = 40) demonstrated better performance of MRI compared to this nomogram. Furthermore, since the nomograms do not include the laterality of T3, MRI may have an additional advantage.

In this study, we used a 1.5T MRI scanner fitted with a body surface coil. We used only 3D T2 images with 0.9-mm isotropic voxels and DW sequences. The European Society of Urogenital Radiologists (ESUR) 2011 recommends 3 mm T2 images acquired in three planes at maximum 0.7 × 0.7 mm in-plane resolution. Furthermore, both dynamic contrast-enhanced (DCE) images and DWI are recommended, while spectroscopy is optional [14].

Since we use 3D T2 acquisition, multiplanar reconstruction is possible without increasing the scanning time. Moreover, we consider 0.9 mm sections with 0.9 mm in-plane resolution to be more important than thicker sections (3 mm) at slightly higher in-plane resolution (0.7 mm). Endorectal coil is also recommended by the ESUR 2011 in order to improve the signal-to-noise ratio, although Fütterer et al. [23] (prospective, *n* = 81) failed to demonstrate improved sensitivity for detecting pT3 disease when comparing endorectal and surface coils. An endorectal coil deforms the prostate gland and causes signal artifacts close to the coil. This region constitutes the majority of the peripheral zone, which is usually the location of the index tumor and EPE. Moreover, 3T MRI is more susceptible to motion artifacts compared to 1.5T MRI. For all these reasons, 3T endorectal MRI may potentially affect MRI staging in a negative direction. In order to decide which MRI method is superior for staging, 1.5T and 3T MRI should be compared in a randomized clinical trial (RCT). However, our results indicate that a simpler protocol on a commonly available 1.5T MRI without DCE images and without endorectal coil may perform equally well or even better compared to the recommended method.

This study is limited in that all MRI examinations were performed by a single radiologist, thus precluding an evaluation of interobserver variability and robustness of the method. We performed a balanced MRI reading when assessing T3, e.g., we did not favor a high specificity or sensitivity. Some previous MRI studies have thrived for a high specificity in order to avoid false-positive T3, since these patients were usually deferred from surgical treatment. This difference may cause difficulties when comparing results. Furthermore, in addition to the direct MRI signs of T3, we used indirect signs, such as tumor volume and tumor capsule length for assessment, because a large tumor is more likely to be associated with T3 [24]. Further analyses and establishing cutoff values are necessary to evaluate the value of MRI for this purpose.

The performance of MRI is highly dependent upon the experience of the radiologist. This may influence the applicability of our results to other institutions with both less and more MRI experience. Variations in the prevalence of pT3 will also influence the legitimacy of comparing results. The high prevalence of pT3 in this study may be attributed to the absence of a PSA screening program in Norway. However, since the material included the MRI arm of a RCT comprising all patients scheduled for surgery, selection bias is ruled out.

The circumferential length of EPE was significantly greater in patients correctly classified as T3 compared to those erroneously classified as T2 (9.5 vs. 6.0 mm). There was also a tendency toward better detection rates in patients with EPE >8 mm. To our knowledge, most other studies stratify detection according to the radial diameter, making comparison difficult [12], and no consensus exists how to quantify pT3 [15]. However, regardless of which method used for the quantification of EPE, the tendencies are the same: MRI detects larger EPE and misses smaller magnitudes [12]. This may be clinically important since extensive EPE is believed to be more associated with positive surgical margins compared to focal.

Moreover, we did not include the location of T3 relative to the NVB's, which is relevant during surgery.

Because a standardized cutting mold was not used for all prostate specimens, inaccurate slice thickness may have influenced the evaluation of T3. When preparing whole-mount sections, the slice thickness was 5–8 mm, and 7-µm sections were obtained from the caudal surface. Additional adjacent sections were prepared if the tumor, or related EPE, could not be properly evaluated. However, if small tumors with EPE were concealed between two whole-mount sections without EPE, this region would most likely be missed. Nevertheless, such small tumors are very unlikely to yield EPE [11]. Furthermore, there is ongoing controversies regarding how to classify T3 disease, and there is significant interobserver variability among pathologists [15].

It may be difficult to distinguish a pT3 from an incisional pT2 in case of positive surgical margins. If the cut surface involves the capsule and there is no visible EPE, it will most likely be classified as pT2 with SM+. On the contrary, if the tumor harbors areas with EPE close to the cut surface, it would be classified as pT3. Nerve-sparing surgery may prevent reliable judgment of EPE since the dissection runs very close to the capsule, and borderline situations may attribute to both false-negative and false-positive MRI.

## Conclusion and future prospects

Magnetic resonance imaging (MRI) detected 72 % of all patients with T3 disease, and the accuracy dropped from 69 to 57 % when considering the laterality of T3.

Thus far, the MRI technique is not yet adequate to meet the increasing demands of accurate diagnosis of locally advanced disease, and the contemporary MRI staging should be carefully interpreted in conjunction with the clinical setting.
